# Symptomatic Cavum Septum Pellucidum and Vergae Cyst: A Case Report

**DOI:** 10.3390/reports8020054

**Published:** 2025-04-23

**Authors:** Elīna Runce, Kalvis Verzemnieks, Kaspars Auslands, Zanda Priede, Tõnu Rätsep, Arturs Balodis

**Affiliations:** 1 Faculty of Medicine, Riga Stradins University, LV-1007 Riga, Latvia; eliinarunce@gmail.com; 2 Institute of Diagnostic Radiology, Pauls Stradins Clinical University Hospital, LV-1002 Riga, Latvia; kalvis.verzemnieks@gmail.com; 3 Department of Radiology, University of Latvia, LV-1007 Riga, Latvia; 4 Clinic of Neurosurgery, Riga East University Hospital, LV-1079 Riga, Latvia; kaspars.auslands@rsu.lv; 5 Department of Neurology and Neurosurgery, Riga Stradins University, LV-1007 Riga, Latvia; zanda.priede@rsu.lv; 6 Department of Neurosurgery, Tartu University Hospital, 50406 Tartu, Estonia; tonu.ratsep@kliinikum.ee; 7 Department of Radiology, Riga Stradins University, LV-1007 Riga, Latvia

**Keywords:** cavum septum pellucidum, cavum vergae, hydrocephalus, neuroendoscopic fenestration

## Abstract

**Background and Clinical Significance**: Cavum septum pellucidum (CSP) and cavum vergae (CV) are anatomical variations that may persist into childhood, adolescence, or adulthood. When these cavities become abnormally large, they are classified as cysts. The mechanism leading to expansion is poorly understood. Although rare, symptomatic CSP and CV cysts can present with a wide range of clinical manifestations. **Case Presentation:** A 20-year-old Caucasian male presented with progressively worsening symptoms over several months including persistent headaches and dizziness. Neurological evaluation showed no abnormalities, with intact cranial nerve function, normal muscle strength, and no signs of paresis. Imaging identified CSP and CV cysts causing obstructive hydrocephalus. MRI findings confirmed progressive cyst enlargement and obstruction of intraventricular foramen. The patient underwent neuroendoscopic fenestration of the cyst with resolution of both hydrocephalus and the symptoms. A CT and MRI scan of the brain performed 12 years before revealed a developmental variant, showing no evidence of cyst formation or ventricular enlargement and without hydrocephalus at that time. This case provides a rare opportunity to observe cyst growth dynamics over time. **Conclusions:** This case presents the importance of recognizing symptomatic CSP and CV cysts as rare but significant causes of obstructive hydrocephalus. The progression from a developmental variant to cyst formation over time illustrates the value of long-term imaging follow-up in such cases. Neuroendoscopic fenestration provided complete resolution of symptoms, demonstrating the effectiveness of surgical intervention in such cases.

## 1. Introduction and Clinical Significance

Cavum septum pellucidum (CSP) and cavum vergae (CV) are midline anatomical variations of the brain that involve cerebrospinal fluid (CSF) spaces located between the lateral ventricles. The septum pellucidum (SP) is a thin, triangular membrane separating the right and left frontal horns of the lateral ventricles, while the CV extends posteriorly from the CSP, forming a continuation of the CSF space [1,2,3].

These structures are considered normal anatomical variations, present in all premature infants and approximately 85% of full-term newborns, with spontaneous closure typically occurring within 3–6 months postnatally [4,5]. However, studies have shown that it persists in approximately 15% of the general adult population [6].

In some cases, these cavities may begin expanding, causing neurological symptoms. The exact mechanism leading to expansion is poorly understood [1]. When these cavities enlarge and form cysts—defined by a CSF space greater than 10 mm in transverse diameter—they can become symptomatic [4,7,8]. The cyst may obstruct the flow of cerebrospinal fluid through the intraventricular foramen, resulting in a range of symptoms such as headache, cognitive impairment, behavioral changes, seizures, dizziness, hydrocephalus, nausea, and vomiting. CSP and CV cysts are typically incidental findings, occurring in approximately 0.04% of imaging studies. Symptomatic cases are rare but can lead to significant morbidity [1,4,5,9].

This unique case report presents the clinical presentation of a symptomatic CSP and CV cyst in a young adult. It is noteworthy that imaging conducted 12 years earlier revealed only a developmental variant with cavum vergae and septum pellucidum, without a fully formed cyst at that time. The condition was successfully treated using neuroendoscopic fenestration, underscoring the effectiveness of this approach in managing such rare but impactful cases.

## 2. Case Presentation

A 20-year-old Caucasian male was admitted to a tertiary university hospital for planned surgery to manage hydrocephalus. The condition had persisted for several months, with progressively worsening symptoms including increasing headaches and dizziness. The patient’s overall condition was stable, with full consciousness, appropriate response, and coherent communication. The Glasgow Coma Scale (GCS) score was 15. No abnormalities in cranial nerve function were detected. Muscle strength in all extremities were intact, with no evidence of paresis.

**Figure 1:**
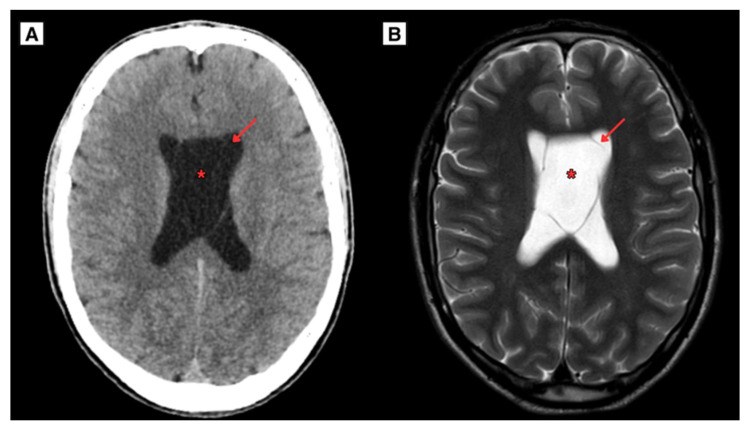
(**A**) CT scan of the brain of a 20-year-old Caucasian male in the axial plane (2024). Demonstrated enlargement of the CSP and CV cysts forming with arched membranous leaves, with the maximum diameter measuring approximately 30 mm, indicating an increase of roughly 17.8 mm. (**B**) MRI scan of the brain revealed CSP and CV cysts and hydrocephalus, presenting with progressively worsening symptoms over several months including increasing headaches and dizziness. The cysts developed from the CSP and CV leaves. **Red arrow**—septum pellucidum wall, **Red asterisk**—cavum septum pellucidum.

**Figure 2:**
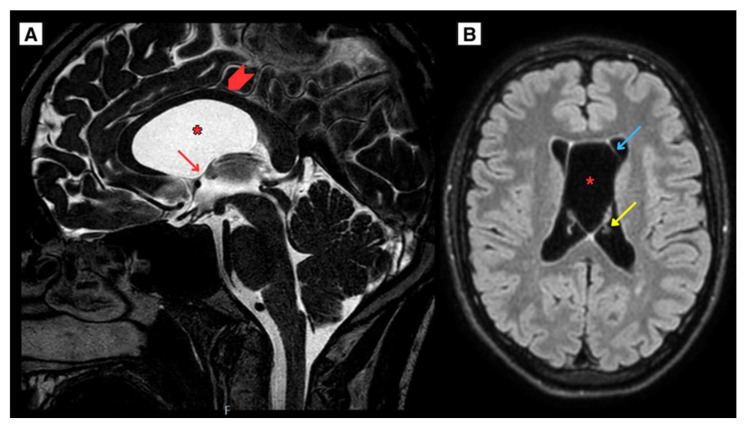
(**A**) T2W sagittal sequence. The corpus callosum was elevated and displaced, with the fornix compressed. No significant optic nerve deformation or impact on the hypothalamus was observed. Wider lateral ventricles with the left temporal horn dilated up to 5 mm. (**B**) T2W-FLAIR axial sequence with the CSP and CV cysts showed the maximum diameter measuring approximately 30 mm covering the intraventricular foramen. **Arrowhead**—corpus callosum, **Red arrow**—intraventricular foramen, **Blue arrow**—cavum septum pellucidum wall, **Yellow arrow**—choroid plexus of lateral ventricle, **Asterisk**—cavum septum pellucidum and cavum vergae cyst.

The CT scan of the 20-year-old patient brain demonstrated the presence of both CSP and CV cyst, representing a dilated ventricles, occlusive hydrocephalus without edema (Figure 1). Follow-up MRI scan of the brain revealed obstructive hydrocephalus with progressive ventricular enlargement (Figure 2).

**Figure 3:**
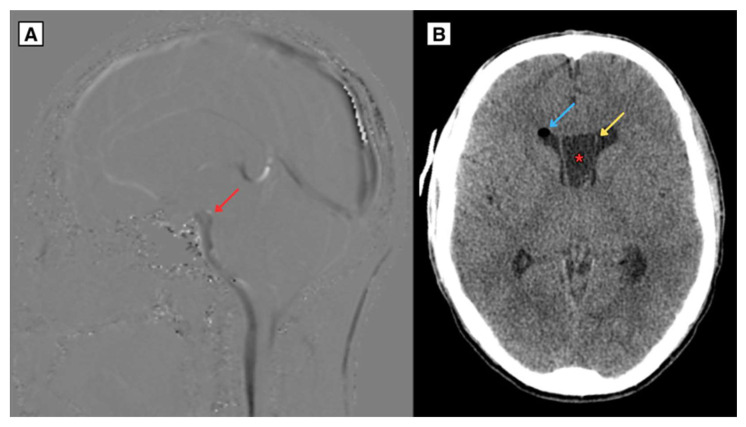
MRI of the brain (**A**) CSF sagittal sequence (2024) of CSP and CV cysts covering the intraventricular foramen resulted in CSF flow disturbances, with almost no visible CSF flow through the cerebral aqueduct and the central spinal canal. Additionally, weakened CSF flow was noted dorsal to the medulla oblongata while increased flow was observed ventrally to the medulla oblongata. (**B**) CT brain scan in axial plane (2024) post endoscopic fenestration of the right side of the CSP cyst revealed brain tissue edema and air present at the surgical access site in the right frontal lobe. The CSP maximum diameter measured approximately 21.2 mm. **Red arrow**—interventricular foramen, **Blue arrow**—pneumocranium, **Yellow arrow**—septum pellucidum wall, **Asterisk**—septum pellucidum cyst.

As the CSP and CV cysts enlarged, they covered the intraventricular foramen, contributing to significant obstruction of the CSF pathways. This resulted in cerebrospinal fluid (CSF) flow disturbances, with almost no visible CSF flow through the cerebral aqueduct and the central spinal canal. Additionally, weakened CSF flow was noted dorsal to the medulla oblongata, while increased flow was observed ventrally to the medulla oblongata (Figure 3). These findings illustrate the physiological consequences of the progressive cyst growth and their role in the development of obstructive hydrocephalus.

The patient underwent neuroendoscopic fenestration of the cyst, which led to the resolution of both hydrocephalus and the symptoms. In collaboration with the Department of Neurosurgery, extensive efforts were made to retrieve and analyze the procedural images. However, due to technological limitations, it was not possible to save or export the imaging data from the procedure. A follow-up CT brain scan one day after endoscopic fenestration of the right side of the CSP cyst revealed brain tissue edema and air present at the surgical access site in the right frontal lobe.

### 2.1. Retrospective Imaging Review

**Figure 4:**
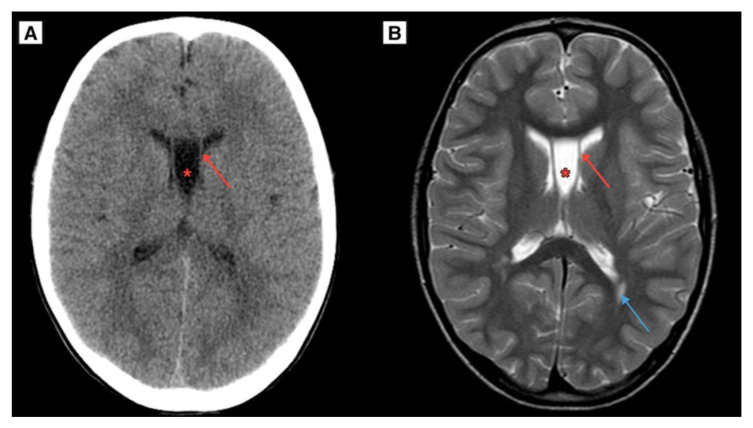
(**A**) A CT scan of the brain in the axial plane, performed on an 8 year-old Caucasian male (2011) demonstrated the developmental variant of CSP and CV, with a maximum diameter of approximately 12.2 mm. (**B**) The same patient’s MRI scan of the brain T2W axial sequence demonstrated a CSP and CV developmental variant. Notably, **Blue arrow**—linear lesion-like focal changes along the posterior parts of the lateral ventricles, which could be caused by previous mild perinatal injury. **Red arrow**—septum pellucidum wall, **Red asterisk**—CSP and CV.

The patient had a CT scan of the brain 12 years ago at the time of a head trauma, which showed no evidence of ventricular enlargement, intracranial cysts, hydrocephalus, or any symptoms. CT brain scan imaging revealed a CSP and CV developmental variant (Figure 4), providing a unique example of evaluating the progression of CSP and CV growth in dynamics. Additionally, an MRI scan of the brain was performed 12 years ago, demonstrating an exceptional case of CSP and CV cyst dynamic progression (Figure 4). Notably, linear lesion-like changes along the posterior parts of the lateral ventricles were observed, which may indicate a possible past mild perinatal injury. In this case, such an injury could represent one of the rarely described potential etiological factors contributing to the development of a CSP cyst over the patient’s lifetime.

### 2.2. A Novel Anatomical Pattern in CSP Cyst Imaging

**Figure 5:**
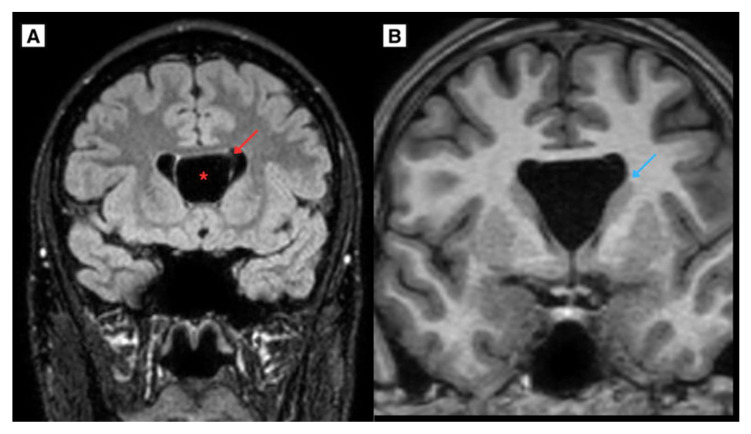
In the MRI of the brain (**A**) T2W, FLAIR MPR coronal sequence with the CSP and CV cysts showed the maximum diameter measuring approximately 30 mm and (**B**) T1, MPR coronal sequence (2024), a distinct feature resembling, according to the authors, the shape of an elephant head was observed. **Red asterisk and red arrow**—CSP and CV cyst, **Blue arrow**—“elephant head sign”.

In the MRI of the brain coronal sequence, a distinct feature resembling, according to the authors, the shape of an elephant head was observed. This remarkable anatomical pattern, due to its similarity to the silhouette of an elephant, could be referred to as the “elephant-head sign”. The adoption of such terminology may enhance the clarity of radiological interpretation and improve communication among clinicians by providing an intuitive reference point for CSP cysts. However, while the introduction of this novel sign is creative and may hold potential as a diagnostic aid, further research involving larger sample sizes is needed to assess its reproducibility and establish its diagnostic utility. In the future, a systematic review or meta-analysis of similar published case reports could provide additional clinical relevance of this proposed sign (Figure 5).

## 3. Discussion

The cavum vergae (CV) is a fluid-filled space situated between the laminae of the septum pellucidum, positioned posterior to an imaginary vertical plane defined by the columns of the fornix. It typically communicates with the cavum septi pellucidi (CSP), and both structures undergo a posterior-to-anterior obliteration, with the cavum vergae closing first, followed by the cavum septi pellucidi [10].

The septum pellucidum (SP) is a thin, triangular, and delicate brain structure situated between the lateral ventricles. Composed of both white and gray matter, it forms part of the limbic system, which is responsible for regulating emotions, behavior, and memory. The SP serves as a connection between the corpus callosum—a major nerve fiber bundle that links the two cerebral hemispheres—and the fornix, a key structure involved in memory function. A frequent anatomical variation of the SP, known as the cavum septi pellucidi, has garnered interest due to its potential links to various neurodevelopmental and psychiatric disorders [6].

The development of the septum pellucidum occurs in parallel with limbic structures such as the corpus callosum, hippocampus, amygdala, and septal nuclei, which are anatomically and functionally interconnected. Variations in septum pellucidum anatomy are thought to arise from disruptions in the embryonic development of these adjacent structures. During fetal growth, the expanding corpus callosum and hippocampus exert pressure on the septal leaflets, promoting their progressive obliteration from posterior to anterior. It is hypothesized that the dysgenesis of these structures disrupts this process, leading to persistent CSP by preventing its normal closure [2].

CSP and CV cysts are potential cerebrospinal fluid (CSF)-filled spaces located between the leaflets of the tela choroidea of the third ventricle [11]. The CSP is bordered superiorly by the crus of the fornices and inferiorly by the tela choroidea of the third ventricle, while the CV extends posteriorly beyond the columns of the fornix [11].

A possible mechanism of cyst enlargement is cerebrospinal fluid transudation from the lateral ventricles. CSF release into the cyst may be carried out by residual embryonic cells of the arachnoid membrane localized in cystic walls. However, this assumption has not been histologically confirmed. Assumptions on the migration of cells capable of CSF release to the cavum septum pellucidum, or transformation of cystic wall cells into those capable of CSF release, were also made. These processes result in transformation of the cavum [5].

A study by Chen et al. involving 19,031 patients aged 0 to 99 years (mean age: 52.6 years) identified CSP and/or CV in 177 cases (0.93%), with 95.5% of these cases exhibiting both CSP and CV [6]. When these cavities become abnormally large, they are referred to as cysts. A CSP cyst is characterized by the presence of cerebrospinal fluid (CSF) between the lateral ventricles, with the cavity walls being arched rather than parallel and separated by a distance of 10 mm or more. Larger cysts are associated with an increased risk of neuropsychiatric symptoms, with severity often correlating to cyst size [4,7,8].

Although typically considered managed conservatively, these cysts can sometimes become symptomatic. In a study by Wang et al., 22 out of 54,000 MRI patients (0.04%) were found to have a dilated CSP cyst [8].

Depending on its communication with the ventricles, CSP cysts are classified as communicating and non-communicating. Secondary communications may be formed by head trauma, surgery, or spontaneous rupture. According to Shaw et al., CSP and CV cysts can be categorized as either incidental (asymptomatic) or pathological (symptomatic). The exact prevalence of symptomatic cases remains unclear [12]. Given the nature of these structures, it is proposed that they may be classified as pseudocysts—fluid-filled spaces that lack a distinct cystic membrane. These formations are typically incidental findings and are unlikely to possess clinical significance unless they enlarge or induce symptoms. Fundamentally, these spaces represent a potential cavity where cerebrospinal fluid (CSF) accumulation occurs due to an insufficiency in the normal flow of CSF, leading to its buildup between the membranous leaflets [12].

CSP and CV cysts are generally considered incidental findings; however, some can become pathological due to specific mechanisms [13]. These include obstruction of the interventricular foramina, leading to hydrocephalus or increased intracranial pressure; compression of the hypothalamic-septal triangle, resulting in neuropsychiatric symptoms; compression of the optic chiasm and associated pathways; and chronic deep venous involvement, which may cause progressive focal neurological deficits. Hypothalamic-septal triangle compression is associated with mental health changes [1,14,15]. The patient reported headaches and dizziness persisting over several months. Imaging revealed displacement of the corpus callosum, compression of the fornix, and closure of the intraventricular foramen, though no significant effect on the optic chiasm was observed. It is more likely that the symptoms are due to the involvement of the hypothalamic-septal triangle.

Although the cyst was congenital, no definitive factors could be identified to explain its symptomatic presentation during late adolescence in this case. One possible contributing factor, based on the imaging findings, is a suspected mild perinatal injury. Small linear lesions observed in the FLAIR sequence along the posterior parts of the lateral ventricles may suggest a remote perinatal insult. However, given the lack of direct supporting data, this hypothesis should be interpreted with caution. Further research and analysis of similar clinical cases are necessary to determine whether subtle perinatal injuries may play a role in predisposing individuals to symptomatic cyst enlargement later in life.

A meta-analysis of 368 patients identified various signs and symptoms associated with CSP and CV cysts including headache (50%), seizures (23.6%), reduced intelligence or delayed psychomotor development (20.1%), mental disorders (15.8%), dizziness, nausea, and vomiting (10.9%), impaired consciousness (9.8%), gait disturbances (9%), visual impairment (8.4%), optic nerve swelling (4.6%), cranial nerve dysfunction (4%), and hydrocephalus (16.6%) [5]. In this case, the primary symptom—headache—aligned with the typical presentation of the condition.

Headaches are the most common and consistent symptom associated with CSP cysts. These headaches are believed to result from intermittent hydrocephalus, which is triggered by positional changes or activities that increase intracranial pressure such as the Valsalva maneuver or straining. CSP cysts are a rare but significant cause of positional headaches that may be reversible. When diagnosing anterior midline cysts in the brain, it is important to differentiate CSP cysts from other conditions including asymmetric cysts in the lateral ventricles where the septum pellucidum is bowed but remains intact, vein of Galen aneurysms, cavum septum arachnoid cysts, and interhemispheric cysts linked to agenesis of the corpus callosum or cavum velum interpositum [3].

A systematic review of 37 articles out of 344 that included 54 patients with symptomatic CSP identified the most commonly performed procedures as endoscopic fenestration (70,9%, n = 39), shunting (18.2%, n = 10), open surgery (5.5%, n = 3), and stereotactic fenestration (1.8%, n = 1). The review concluded that open surgery and shunting were associated with less favorable outcomes compared with endoscopic or stereotactic approaches such as frontal, parietal, or transcavum fenestration. Notably, half of the patients treated with shunting experienced recurrence of the condition [4]. Our patient underwent neuroendoscopic fenestration of the cyst with resolution of both hydrocephalus and the symptoms.

Endoscopic fenestration typically involves creating a burr hole craniotomy, most often in the right frontal region, to establish communication between the cyst and the lateral ventricles. Alternative approaches include parietal cystostomy or direct transcavum interforniceal endoscopic fenestration. While no significant differences in outcomes have been observed among these techniques, they are all associated with a reduction in cyst size, offering a minimally invasive option with lower recurrence rates and improved clinical outcomes [1,4,16,17].

## 4. Conclusions

This study provided a rare progression of CSP and CV cysts from a developmental variant to symptomatic obstructive hydrocephalus, as documented through long-term imaging. Neuroimaging played a crucial role in diagnosing the condition and tracking the cyst growth dynamics over time. One possible contributing factor, considering the imaging findings in this case, could be a suspected mild perinatal injury. Further research is needed to analyze similar clinical cases and determine whether subtle perinatal injuries may predispose certain individuals to symptomatic cyst enlargement. Neuroendoscopic fenestration proved effective, underscoring the importance of timely intervention in managing these uncommon conditions.

## Data Availability

The original contributions presented in the study are included in the article, further inquiries can be directed to the corresponding author.
